# Factors Affecting the Cervical Cancer Screening Behaviors of Japanese Women in Their 20s and 30s Using a Health Belief Model: A Cross-Sectional Study

**DOI:** 10.3390/curroncol29090494

**Published:** 2022-08-31

**Authors:** Zhengai Cui, Hiromi Kawasaki, Miwako Tsunematsu, Yingai Cui, Masayuki Kakehashi

**Affiliations:** 1 Graduate School of Biomedical and Health Sciences, Hiroshima University, Hiroshima 734-8551, Japan; 2 School of Nursing, Guangdong Medical University, Dongguan 523808, China

**Keywords:** cervical cancer screening, Japanese young women, health belief model, a cross-sectional study

## Abstract

In recent years, the incidence and mortality rates of cervical cancer (CC) have increased among young women. Cervical cancer screening (CCS) is crucial to reducing the incidence and mortality of CC in a country such as Japan, where it is challenging to raise HPV vaccination rates. The purpose of this study was to identify psychological and personal characteristics relating to CCS participation among young people by using the Health Belief Model (HBM). For this cross-sectional study, an internet survey was conducted between February–March 2018. Based on HBM and personal characteristics, χ2 tests and logistic analyses were used to identify factors influencing CCS. Responses obtained from 816 women in their 20s and 30s were used in the analysis. For HBM-based psychological characteristics, the odds ratios were significantly higher for “cues to participation in screening” and “barriers to participation at the time of cancer screening”, while “barriers to participation before cancer screening” showed significantly lower odds ratios. On the other hand, it was found that the presence of children and having regular health checkups affected the attributes of screening that were significant for decision-making. Therefore, it is important to create proactive measures to encourage younger women to undergo medical examinations.

## 1. Introduction

Worldwide, cervical cancer (CC) is the fourth most common cancer among women and the fourth leading cause of death among women due to cancer [1]. In Japan, on the other hand, about 12,000 people are affected annually, and about 3500 people die due to this disease [2]. In recent years, morbidity and mortality rates have been increasing more rapidly among those in their 20s to 30s compared to other age groups due to changes in sexual activity rates among young people [3,4].

People in their 20s and 30s have different levels of CC morbidity and mortality rates than younger or older adults. As these individuals still have long life expectancies, make significant contributions to the economy, and play an important role in supporting their families, as a consequence, the impact is still huge [5]. Therefore, targeted prevention is needed to reduce CC morbidity and mortality rates in this age group.

The CC is caused by persistent infection with the human papillomavirus (HPV) [6,7,8]. It has been shown that CC develops through cervical intraepithelial neoplasia (CIN), a precancerous lesion. Early detection and treatment can be achieved through primary prevention (HPV vaccination) and secondary prevention (cervical cancer screening: CCS).

In the United States and Scotland, where HPV vaccination was adopted as a national program for primary prevention many years ago, the HPV infection rate among women in their 20s has been significantly reduced among vaccinated individuals [9,10]. In Japan, on the other hand, the HPV vaccine was approved at the end of 2009 and became part of routine immunization coverage under the Immunization Law in April 2013. However, in June of the same year, the “recommendation” of HPV vaccination was discontinued due to reports of adverse reactions such as orthostatic intolerance, chronic regional pain syndrome (CRPS) [11], and cognitive dysfunction [12] after HPV vaccination. As a result, the HPV vaccination coverage dropped sharply from over 70% among women born between 1994 and 1999 to less than 1% among women born after 2002 [13,14,15]. This was mainly reported by the Japanese mass media as being due to the possibility of adverse events caused by HPV vaccination, and although the government subsequently began to recommend it again, the vaccination rate has not yet returned to the level it was before the reports of adverse effect [13].

On the other hand, as a form of secondary prevention in Japan, it has been recommended since 2004 that asymptomatic women over 20 years of age undergo CCS (Cytology alone, HPV testing, and Combined cytology and HPV testing) regularly every 2 years [16,17,18]. The “Basic Plan for the Promotion of Cancer Control” formulated in 2007 set the goal of increasing the screening uptake rate to more than 50% within five years [19], and since FY2009, the “Women-specific Cancer Screening Promotion Project”, which includes the distribution of free coupons, has been actively implemented. However, the results of the National Cancer Survey in 2019, conducted 9 years later, showed that the CCS participation rate was 43.7% [20]. The increase in the incidence of CC among young people thus remains a concern. The factors affecting the CCS behaviors in young people and other age groups, as identified in other studies, were differences in health care policies, inadequate public health education, socio-cultural health beliefs, personal difficulties, and a great lack of knowledge about the necessity of screening [21,22,23]. Therefore, based on the results of previous studies, it is necessary to identify the reasons why people might not participate in CCS and create a response plan for young people for whom HPV vaccination has been stagnant.

In order to increase the rate of participation in cancer screening, it is necessary to analyze the psychological characteristics that influence screening participation. In Europe and the United States, many studies have been conducted with the aim of improving cancer screening uptake rates using theories of applied behavioral science [24,25]. Therefore, we considered the Transtheoretical Model of Behavior Change [26] and the Health Belief Model (HBM). However, in order to determine individuals’ perceptions of threats posed by health problems, the benefits of avoiding threats, and factors influencing decision-making, this research survey was conducted using HBM.

The HBM, proposed by Rosenstock [27] in the United States, is a well-known theoretical model that explains the relationship between preventive health care practices and psychological attitudes. According to the first HBM theory, the interaction between the factors of the perceived risk of contracting a particular disease (perceived susceptibility), perceived seriousness of the disease contracted (perceived seriousness), perceived benefit of the practice to prevent that disease (perceived benefit), and perceived barriers to prevention determines the success of specific preventive health practice. In Japan, Hata [28] proposed a revised HBM based on these basic concepts, which have mainly been used in Europe and the United States [29,30,31,32,33,34]. In Japan, there have been reports of screening behaviors associated with group health screening [35] and gastric cancer screening [36], but unfortunately, there have been few applied studies focusing on young adults’ CCS behaviors. Therefore, research based on this theory seems necessary in order to understand the psychological characteristics of young adults’ CCS behavior.

This study aimed to identify the factors of psychological and personal characteristics that influence the behavior of young people regarding receiving CCS by using HBM.

## 2. Materials and Methods

### 2.1. Study Design

A cross-sectional study was used to accomplish the objectives of this study; an internet survey was conducted between February 2018 and March 2018.

### 2.2. Data Collection

The study, conducted via an internet survey, was designed to investigate in a short period of time the intentions of people who were not screened for cancer in a wide geographic area. At the same time, there are two verification standards for internet survey companies: 1. Organizations providing services; 2. External certification in accordance with national standards.

In order to reduce the burden of the respondents, starting from the creation of the online questionnaire, we participate in the design and revision of the questionnaire from time to time. Before the actual survey, in order to ensure the credibility of the participants’ responses, we actually operated the online questionnaire and measured the shortest time, and set the questionnaires whose answer time is less than the shortest time as meaningless data.

A sampling frame was used for “registered monitors” held by the survey contractor (internet research company, Hiroshima, Japan). Age groups (20s, 30s, 40s, 50s, and 60s) were set, and samples were extracted for each category according to the population–population ratio (see 2015 census). The survey was terminated when the target sample for each category was reached. A sample of approximately 3000 persons was deemed adequate.

In this study, the internet research company made the survey target users, and a survey request e-mail was sent out. The survey targets accessed the URL provided in the e-mail and responded to the survey.

The internet survey inclusion criteria were, as of 1 April 2019, (1) women living in Japan, (2) being aged 20–69 years, and (3) having agreed to complete the questionnaire.

However, in this study, data were extracted from an internet survey and analyzed and studied. The extraction criteria were: (1) only those aged 20–30 years were selected according to the purpose of the study; (2) only those who had participated in cervical cancer screening and those who had never participated in cancer screening were selected because of the difference in screening site and age between breast cancer screening and cervical cancer screening [37]; and (3) only those who had no history of cancer were selected because screening attitudes were related to medical history [38].

The questionnaire was constructed based on previous studies [39,40,41,42] and included personal characteristics: age (20–24 years, 25–29 years, 30–34 years, and 35–39 years), marital status (married, single), children, household composition (single, 2-person household, 2-generation family, 3-generation family, and others), employment status (self-employed, regular employment, parttime job, students, housewife, and unemployed), medical insurance (association health insurance, union health insurance, mutual aid association, national health insurance, national health insurance association, unknown, and others), body mass index (BMI) (lean, normal, and obese), current health condition (bad, slightly bad, normal, slightly good, and good), and history of family cancer (Supplementary Table S1).

The CCS participation status: history of screening participation in the past 2 years, types of participation (population-based, workplace-based, individual complete physical examination/hospital visit, and others), and reasons for non-participation (busy, healthy, anxious about the results, did not know about cancer screening, never had a chance to have a cancer screening, forgot to take the test, not old enough to have a checkup, too much trouble, and others) was extracted from the public opinion survey on cancer control [43] (Supplementary Table S2).

On the other hand, psychological characteristics of HBM were set based on previous studies [44,45,46,47]. The HBM items survey consisted of 27 questions components; each item was answered on 5-point Likert scale (1: strongly disagree, 2: disagree, 3: neither agree nor disagree, 4: agree, 5 strongly agree). The higher scores on each of the items indicated that participation in screening was perceived as more beneficial (Supplementary Table S3).

### 2.3. Statistical Analysis

First, in order to analyze the association between personal characteristics and screening behavior, χ2 tests were conducted to obtain geo-demographic information, lifestyle information, and personal characteristics. Next, based on the results of the χ2 test and the seven constructs from the HBM, logistic regression analysis was conducted with factors significantly associated with screening behavior as independent variables and screening behavior (women who had undergone cervical screening and women who had not) as dependent variables, and all risk factors were considered in the model inputs unless there was evidence of collinearity. The results of the analysis were presented as (adjusted) odds ratios (ORs) and 95% confidence intervals (CIs).

A factor analysis was conducted on 27 variables based on the HBM in order to confirm their structure in the HBM study subjects used as explanatory variables in the logistic regression analysis. The results showed that all the variables were categorized into seven factors based on the HBM model in the survey subjects as well. Cronbach’s alpha coefficient, a measure of internal consistency among the questions comprising each scale, was 0.818 for “seriousness of cancer”, 0.820 for “benefits of cancer screening”, 0.872 for “the importance of cancer screening”, 0.681 for “cues to participation in screening”, 0.699 for “susceptibility to cancer”, 0.812 for “barriers to participation at the time of cancer screening”, and 0.752 for “barriers to participation before cancer screening”. A Cronbach’s alpha coefficient greater than 0.748 was usually considered to be a reasonable representation of the construct variables based on the factors obtained.

Statistical analysis was performed using R version 4.0.2. *p* < 0.05 was considered statistically significant.

### 2.4. Ethics Issues

This survey was conducted with the approval of the Hiroshima University Epidemiology Ethics Committee (Approval No. E-1081-1). Respondents were informed that no personally identifiable information would be collected, that it was up to them whether or not to participate in study, and that they would not be harmed if they did not respond.

## 3. Results

A total of 3249 people responded to the questionnaire. In accordance with the exclusion criteria, (1) 1970 persons not in their 20s or 30s, (2) 255 persons who had undergone breast cancer screening, and (3) 208 persons with a medical history of cervical cancer were excluded, leaving a total of 816 persons in the analysis. Of these, 32 (3.9%) lived in Hokkaido, 60 (7.4%) people in Tohoku, 303 (37.1%) Kanto Koshinetsu, 123 (15.1%) in Tokai Hokuriku, 130 (15.9%) in Kinki, 70 (8.6%) in Chugoku Shikoku, and 98 (12.0%) in Kyushu Okinawa. Of those analyzed, 321 (39.3%) were screened, with a mean age of 30.85 years (SD ± 14.88), and 495 (60.7%) were not screened, with a mean age of 30.24 years (SD ± 15.35).

### 3.1. Factors Affecting Participation in CCS

After the association between the personal characteristics and screening behaviors had been assessed using the χ2 test, significant associations were determined to be age, marital status, children, work status, medical insurance, and regular health checkups (Table 1).

The screening rates of women who had undergone screening were in the order of 45.0% (95 women) in those in their 30–34s, 44.4% (130) in those in their 25–29s, 37.2% (80) in those in their 35–39s, and 16.5% (16) in those in their 20–24s, 51.7% (238) in those who were married, and 23.3% (83) in those who were single. The status of participation in CCS is shown according to each attribute in Table 1.

### 3.2. Status of Participation in CCS

Table 2 shows the participation in CCS by each demographic characteristic: 9 (56.3%) women in the 20–24 age group, 52 (40.0%) in the 25–29 age group, and 51 (53.7%) in the 30–34 age group participated in the individual complete physical examination/hospital visit. In this population-based study, 37 (46.3%) women between the ages of 35–39 years participated. Of these, 7 (50.0%) self-employed people, 49 (35.3%) people in regular employment, 4 (80.0%) students, and 55 (52.4%) housewives participated in individual complete physical examinations/hospital visits. More than half of the women who have parttime job (*n*= 28 (57.1%)) and unemployed (*n* = 5 (55.6%)) participated in this population-based study.

Table 3 shows not participating in CCS by age, employment status, and medical insurance: the age group, 32 (39.5%) people in the 20–24 age group selected “I don’t think I am old enough to have a checkup” as their reason for not participating in the screening. On the other hand, 73 (44.8%) in the 25–29 age group, 42 (36.2%) in the 30–34 age group, and 46 (34.1%) in the 35–39 age group gave their most common reason for not participating in the screening as “Because I never had a chance to have a cancer screening”.

By employment status, 6 (50.0%) self-employed workers, 81 (40.3%) full-time workers, 37 (35.9%) parttime workers, and 43 (43.0%) full-time housewives were unable to participate in the screening because “because I never had a chance to have a cancer screening”. For 21 students (51.2%), the reason was “I don’t think I am old enough to have a checkup”. For nine unemployed workers (23.7%), the reason was “because I forgot to take the test”.

By medical insurance, 74 (44.3%) of the respondents were from the Association Health Insurance, 31 (50.0%) from Union Health Insurance, 8 (20.5%) from the Mutual Aid Association, 52 (30.8%) from the National Health Insurance, and 11 (35.5%) from the National Health Insurance Association did not participate in CCS “Because I never had a chance to have a cancer screening”.

### 3.3. Psychological and Personal Characteristics affecting Participation in CCS

Table 4 shows the results obtained for the psychological characteristics of the screened group based on the HBM; the odds ratios were significantly higher for “cues to participation in screening” was 1.33 (95% CI, 1.05–1.69) (*p* = 0.020) and “barriers to participation at the time of cancer screening” was 1.40 (95% CI, 1.08–1.81) (*p* = 0.012), whereas the odds ratio was significantly lower for “barriers to participation before cancer screening” was 0.32 (95% CI, 0.23–0.45) (*p* < 0.001). Among the personal characteristics, those aged 25–29 was 2.22 (95% CI, 1.37–3.60) (*p* = 0.001), who had children was 2.21 (95% CI, 1.15–4.25) (*p* = 0.018), and who had regular health checkups were 2.32 (95% CI, 1.38–3.90) (*p* = 0.001) were significantly associated with screening behaviors.

## 4. Discussion

In order to improve the CCS participation rate among young people, this study combined subjective (reasons for participation/non-participation in screening) and objective (χ2 test and logistic analysis) perspectives for the analyzed subjects in order to better understand the CCS screening participation status of young people and develop promotional measures.

The χ2 test results indicated that age, marital status, child status, employment status, medical insurance, and health awareness were associated with CCS visit behavior. This result is consistent with that of previous studies [39,40,41,42].

Following the Great East Japan Earthquake in 2011, people affected by the disaster continued to be mentally and economically unstable and unable to maintain their health [48,49,50], and as a result, CCS participation rates in coastal areas were significantly lower than in other areas [51]. Areas affected by disasters are predicted to take 10 years to recover [52]. However, this study found no significant differences in population size or residential area. Thus, it is believed (as of 2018) that screening participation rates among young people are influenced by employment status and the nature of the community rather than by region of residence.

The rate of participation of younger people in CCS was still low. The Japanese government’s “Basic Plan for the Promotion of Cancer Control” target of increasing the screening participation rate to 50% or more within five years has not yet been achieved [19]. In addition, the WHO recommendation that countries achieve targets for HPV vaccination coverage of 90%, cervical cancer screening coverage of 70%, and cervical lesion treatment coverage of 90% by 2030 [53] was also difficult to achieve. Therefore, it is very important to identify the psychological and personal characteristics that influence how young people behave when receiving CCS in other to improve the rate of participation of young people in CCS.

The aggregate results of CCS participation/reasons for not participating showed that only a small number of people in the 20–24 age group participated in CCS as a result of a physical exam or hospital visit. The reason for a large number of people not participating was that they did not consider themselves to be at an age where they needed to participate in CCS. Women in this age group are likely to have little experience with serious illnesses and are overconfident about their health. In addition to this, we found that there was a lack of available information about CC and CCS and that gathering information about medical examination behavior in the absence of subjective symptoms was seen as a hassle, reducing the willingness of this group to undergo medical checkups [54]. Therefore, people in this group did not have the opportunity to correct their erroneous perceptions of CCS and probably viewed CC as a disease that had nothing to do with them. In addition, due to the lack of information on CC and CCS, there seemed to be deep-seated resistance to seeing an obstetrician/gynecologist, including resistance to being mistaken for being pregnant, anxiety about contact with acquaintances, and discomfort about being seen with a pregnant woman. Therefore, it is necessary to focus on overcoming resistance to screening among 20–24-year-olds [55], actively provide them with information on CC and CCS, and encourage them to participate in cancer screening from appropriate age.

On the other hand, women in the 25–29 and 30–34 age groups are in the first marriage and first childbearing age groups [56] and are more likely to be employed due to their entry into the workforce [57]. Along with this, since they have employee insurance, they are more likely to participate in CCS through workplace-based screening. In Japan, there are four main types of employee insurance such as “Association health insurance,” “Union health insurance (for employees of large companies and their dependents),” “Mutual aid association (for public employees and their dependents),” and “National health insurance association (for doctors, construction workers and their dependents)”.

However, we found that more women in the 25–29 and 30–34 age groups participated in CCS through physical examinations and hospital visits instead of workplace examinations. The main reason for not participating in CCS screening as they did not have the chance to undergo CCS screening. Because of this, even if they miss the chance to be screened at work, they are encouraged to be screened for CCS by providing the chance to do so. According to a previous study, 60.9% of those who do not have the opportunity to receive checkups at work do not receive community checkups [58], so it is considered necessary to take measures to direct those who do not have the opportunity to receive checkups at work to places in the community where they can receive checkups.

In addition, women in the 35–39 age group mainly participated in CCS screening mainly through community checkups. Women in this age group are more concerned about work than about family because they are busy at work and tend to worry less about childbirth and childcare. Therefore, it is likely that the opportunity to participate in CCS checkups through the workplace will be overlooked. It would be important to increase the opportunities for this age group to participate in CCS examinations at work.

This logistic analysis revealed the factors promoting CCS screening (children, regular health checkups, cues to participation in screening, and barriers to participation at the time of cancer screening) and factors acting as obstacles (barriers to participation before cancer screening). Therefore, the following factors were considered to be important in promoting CCS: recommendations to undergo women’s cancer screening from close family members, close friends, and acquaintances; recommendations made by doctors at regular health checkups; a reduction in the number of male doctors and staff at hospitals; and a reduction in discomfort and pain during screening due to improved technology and screening knowledge, as well as a reduction in shame during screening. On the other hand, the following factors were identified as obstacles: forgetting to go for checkups, concerns about time and cost, and uncertainty about where to go for checkups. Based on facilitators and inhibitors, we believe that effective measures can be developed to increase participation in CCS screening in the future.

A summary of results and points to consider when recommending medical examinations are shown in Table 5.

In Japan, CCS methods include Cytology alone, HPV testing, and Combined cytology and HPV testing. The target ages for each method were 20–69 years, 30–60 years, and 30–60 years, respectively. Preliminary studies suggest a high incidence of cancer in women under the age of 25 and a high regression rate of CIN2 in this age group; CCS is not recommended for women under the age of 25 [59]. On the other hand, every CCS method is accompanied by the occurrence of false positives [18]. Due to the impact of false positives on the psychological burden, production, and parenting of CCS participants, it is suggested that the target age group of CCS should be changed from 20–39 years to 30–39 years. In addition, paying attention to CCS participants and their children can develop the habit of actively participating in CCS, considering that women in the 30–34 age group are the main focus group. In addition, measures A to C were developed to increase the participation rate of CCS.

A. Increase opportunities for medical checkups by coordinating community and workplace medical checkups.

Large-scale establishments have dedicated doctors and medical personnel to encourage employees to undergo health checkups, but small- and medium-scale establishments do not have such dedicated medical personnel and may not be able to provide adequate health management for their employees. Therefore, it is considered necessary to actively inform working women who have few opportunities to receive health checkups at their workplaces that they can receive health checkups in town and encourage them to do so.

B. Self-collected cytological diagnosis reduces the burden at the time of screening.

Self-collection cytology is recommended for those who resist physician-collected cytology due to embarrassment. However, self-collection cytology may not lead to the collection of a sufficient number of cells from the cervix, may result in an inappropriate specimen, or may not result in cells being collected from the SCJ (squamous-cylinder-epithelial junction), a favorable site for CC, resulting in lesions being missed. The CCS guideline [18] points out that this method is suboptimal as a screening method because it may not lead to the collection of a sufficient number of cells from the cervix and may result in the collection of an inappropriate specimen. However, there is little evidence of problems relating to the application of this method.

In recent years, COVID-19 has caused an increasing number of people to refrain from undergoing cancer screening, and there is concern that there are people with cancer who have not undergone screening [60]. Encouraging people to return for screening by enabling them to perform self-sampling cytological diagnosis appears to be a promising countermeasure to this situation. The peak age of CC incidence coincides with the periods of pregnancy, childbirth, child-rearing, and career development. The prevention of CC is recognized as an issue deserving national priority. Sending self-collection kits has been shown to be more effective than sending written reminders to undergo health checkups in inducing groups of people who have not yet been examined to undergo checkups [61]. Therefore, this was considered to be the most effective method for those who wanted to participate in CCS but were unable to do so. However, self-collection of cytology is a major decision that must consider existing infrastructure, political will and commitment to implementation, and cultural aspects of the target population [21,22,23]. Therefore, applying the results of this study to countries other than Japan will depend on the actual situation in each country.

C. Education and knowledge dissemination in CCS to reduce the burden prior to screening.

Education and knowledge included knowledge relating to the characteristics of CC disease, the incidence of cervical cancer by age, and how and where to participate in community screening if workplace screening was not available.

On the other hand, it is considered better to use a video rather than a pamphlet to disseminate basic knowledge on self-administered cytological diagnosis, self-examination methods, and points to note during the examination. It is also necessary to devise a screening tool for self-collection cytology, where the depth of insertion should be clarified with a red line.

## 5. Limitations

There are several limitations to this study. First, because this is a cross-sectional survey, we could only determine associations among variables and could not examine causal relationships among variables. Second, because it was an online survey, in some cases, the attributes of respondents registered as monitors may be biased in some cases. Although the bias of survey targets can be adjusted through screening, it is difficult to ensure the reliability and authenticity of the registered attributes themselves. Another issue is that it is impossible to fully confirm the identity of respondents; for example, it is impossible to prevent respondents who are not the actual respondents from answering the survey, so it was not possible to delve deeply into the needs of 20–30-year-olds regarding cancer screening. Therefore, we plan to employ a mixed qualitative and quantitative survey methodology to validate the results of this study. Finally, HBM theory suggests that the direct determinants of preventive health practices are psychological characteristics and attitudes, called health beliefs, while background factors and personal characteristics are indirect modifiers related to behavior via psychological attitudes. However, this study examined only the main effects of each psychological factor and did not examine any potential interactions occurring among these psychological factors. Future studies should analyze in more detail the correlations between HBM screening behavior and background factors and psychological characteristics.

## 6. Conclusions

We examined psychological and personal characteristics influencing CCS participation using χ2 tests and logistic analyses for the subjects in their 20s and 30s. The results obtained showed that the odds ratios for psychological characteristics based on HBM were significantly higher for “cues to participation in screening” and “barriers to participation at the time of cancer screening”, while, conversely, the odds ratio was significantly lower for “barriers to participation before cancer screening”. On the other hand, it was found that the presence of children and having regular health checkups affected the attributes of screening that were significant for decision-making. It is important to create proactive measures to encourage people in the 30–34 age group to undergo medical examinations. Specific measures included (1) increasing opportunities for undergoing screening through collaboration between community and workplace screening, (2) reducing the burden at the time of screening through the implementation of self-collection cytology, and (3) reducing the burden prior to screening through the dissemination of education and knowledge.

## Figures and Tables

**Table 1:** Associations between CCS behaviors and personal characteristics (Statistically significant results).

Characteristic	Total (*n* = 816)	Screened (*n* = 321)	Unscreened (*n* = 495)	*p*-Value *
Age				<0.001
20–24	97 (11.9%)	16 (16.5%)	81 (83.5%)	
25–29	293 (35.9%)	130 (44.4%)	163 (55.6%)	
30–34	211 (25.9%)	95 (45.0%)	116 (55.0%)	
35–39	215 (26.3%)	80 (37.2%)	135 (62.8%)	
Marital Status				<0.001
Married	460 (56.4%)	238 (51.7%)	222 (48.3%)	
Single	356 (43.6%)	83 (23.3%)	273 (76.7%)	
Children				<0.001
Yes	346 (42.4%)	187 (54.0%)	159 (46.0%)	
No	470 (57.6%)	134 (28.5%)	336 (71.5%)	
Household composition				0.001
Single	145 (17.8%)	37 (25.5%)	108 (74.5%)	
2-person household	163 (20.0%)	82 (50.3%)	81 (49.7%)	
2-generation family	422 (51.7%)	169 (40.0%)	253 (60.0%)	
3-generation family	59 (7.2%)	23 (39.0%)	36 (61.0%)	
Others	27 (3.3%)	10 (37.0%)	17 (63.0%)	
Employment status				<0.001
Self-employed	26 (3.2%)	14 (53.8%)	12 (46.2%)	
Regular employment	340 (41.7%)	139 (40.9%)	201 (59.1%)	
Parttime job	152 (18.6%)	49 (32.2%)	103 (67.8%)	
Students	46 (5.6%)	5 (10.9%)	41 (89.1%)	
Housewife	205 (25.1%)	105 (51.2%)	100 (48.8%)	
Unemployed	47 (5.8%)	9 (19.1%)	38 (80.9%)	
Medical insurance **				<0.001
Association health insurance	319 (39.1%)	152 (47.6%)	167 (52.4%)	
Union health insurance	107 (13.1%)	45 (42.1%)	62 (57.9%)	
Mutual aid association	75 (9.2%)	36 (48.0%)	39 (52.0%)	
National health insurance	233 (28.6%)	64 (27.5%)	169 (72.5%)	
National health insurance association	50 (6.1%)	19 (38.0%)	31 (62.0%)	
Unknown	20 (2.5%)	3 (15.0%)	17 (85.0%)	
Others	12 (1.5%)	2 (16.7%)	10 (83.3%)	
Medical consultation				<0.001
Yes	198 (24.3%)	103 (52.0%)	95 (48.0%)	
No	618 (75.7%)	218 (35.3%)	400 (64.7%)	
Are you taking care of your own health				<0.001
Not careful at all	22 (2.7%)	5 (22.7%)	17 (77.3%)	
Not very careful	134 (16.4%)	38 (28.4%)	96 (71.6%)	
Cannot say either way	186 (22.8%)	62 (33.3%)	124 (66.7%)	
Sometimes very careful	365 (44.7%)	159 (43.6%)	206 (56.4%)	
Always very careful	109 (13.4%)	57 (52.3%)	52 (47.7%)	
What to pay attention to for health				
Pay attention to diet				0.001
Yes	495 (60.7%)	218 (44.0%)	277 (56.0%)	
No	321 (39.3%)	103 (32.1%)	218 (67.9%)	
Have regular health checkups				<0.001
Yes	136 (16.7%)	94 (69.1%)	42 (30.9%)	
No	680 (83.3%)	227 (33.4%)	453 (66.6%)	
Avoid stress				0.028
Yes	282 (34.6%)	126 (44.7%)	156 (55.3%)	
No	534 (65.4%)	195 (36.5%)	339 (63.5%)	
The most feared disease				0.048
Cancer	336 (41.2%)	140 (41.7%)	196 (58.3%)	
Heart disease	51 (6.3%)	24 (47.1%)	27 (52.9%)	
Brain Attack	105 (12.9%)	51 (48.6%)	54 (51.4%)	
Pneumonia	6 (0.7%)	1 (16.7%)	5 (83.3%)	
Diabetes	54 (6.6%)	15 (27.8%)	39 (72.2%)	
Liver disease	10 (1.2%)	4 (40.0%)	6 (60.0%)	
Dementia	83 (10.2%)	27 (32.5%)	56 (67.5%)	
Depression	53 (6.5%)	19 (35.8%)	34 (64.2%)	
None	104 (12.7%)	32 (30.8%)	72 (69.2%)	
Others	14 (1.7%)	8 (57.1%)	6 (42.9%)	
Influenza vaccination				0.001
Vaccinated every year	218 (26.7%)	106 (48.6%)	112 (51.4%)	
Sometimes vaccinated	144 (17.6%)	64 (44.4%)	80 (55.6%)	
Vaccinated by chance	49 (6.0%)	20 (40.8%)	29 (59.2%)	
Not vaccinated	306 (37.5%)	101 (33.0%)	205 (67.0%)	
Thinking vaccination is useless	99 (12.1%)	30 (30.3%)	69 (69.7%)	
Private medical insurance				0.001
Yes	400 (49.0%)	182 (45.5%)	218 (54.5%)	
No	416 (51.0%)	139 (33.4%)	277 (66.6%)	

* χ^2^ test. ** Employee insurance mainly includes “Association health insurance (for employees of small and medium-sized companies and their dependents),” “Union health insurance (for employees of large companies and their dependents),” “Mutual aid association (for public employees and their dependents),” and “National health insurance association (for doctors, construction workers and their dependents)”. Regional insurance includes “National health insurance (for people who are not covered by employee insurance, such as the self-employed and unemployed)”.

**Table 2:** Status of participation in CCS.

Characteristic	Total (*n* = 321)	Population-Based (*n* = 127)	Workplace-Based (*n* = 42)	Individual Complete Physical Examination/Hospital Visit (*n* = 137)	Others (*n* = 15)
Age					
20–24	16 (5.0%)	5 (31.3%)	1 (6.3%)	9 (56.3%)	1 (6.3%)
25–29	130 (40.5%)	51 (39.2%)	19 (14.6%)	52 (40.0%)	8 (6.2%)
30–34	95 (29.6%)	34 (35.8%)	5 (5.3%)	51 (53.7%)	5 (5.3%)
35–39	80 (24.9%)	37 (46.3%)	17 (21.3%)	25 (31.3%)	1 (1.3%)
Employment status					
Self-employed	14 (4.4%)	6 (42.9%)	1 (7.1%)	7 (50.0%)	0 (0.0%)
Regular employment	139 (43.3%)	46 (33.1%)	35 (25.2%)	49 (35.3%)	9 (6.5%)
Parttime job	49 (15.3%)	28 (57.1%)	1 (2.0%)	19 (38.8%)	1 (2.0%)
Students	5 (1.6%)	1 (20.0%)	0 (0.0%)	4 (80.0%)	0 (0.0%)
Housewife	105 (32.7%)	41 (39.0%)	4 (3.8%)	55 (52.4%)	5 (4.8%)
Unemployed	9 (2.8%)	5 (55.6%)	1 (11.1%)	3 (33.3%)	0 (0.0%)
Medical insurance *					
Association health insurance	152 (47.4%)	55 (36.2%)	25 (16.4%)	64 (42.1%)	8 (5.3%)
Union health insurance	45 (14.0%)	12 (26.7%)	7 (15.6%)	21 (46.7%)	5 (11.1%)
Mutual aid association	36 (11.2%)	14 (38.9%)	1 (2.8%)	20 (55.6%)	1 (2.8%)
National health insurance	64 (19.9%)	36 (56.3%)	6 (9.4%)	22 (34.4%)	0 (0.0%)
National health insurance association	19 (5.9%)	7 (36.8%)	3 (15.8%)	8 (42.1%)	1 (5.3%)
Unknown	3 (0.9%)	2 (66.7%)	0 (0.0%)	1 (33.3%)	0 (0.0%)
Others	2 (0.6%)	1 (50.0%)	0 (0.0%)	1 (50.0%)	0 (0.0%)

* Employee insurance mainly includes “Association health insurance (for employees of small and medium-sized companies and their dependents),” “Union health insurance (for employees of large companies and their dependents),” “Mutual aid association (for public employees and their dependents),” and “National health insurance association (for doctors, construction workers and their dependents)”. Regional insurance includes “National health insurance (for people who are not covered by employee insurance, such as the self-employed and unemployed)”.

**Table 3:** Reasons for not participating in CCS by age, employment status, and medical insurance.

Characteristic	Total (n = 495)	Busy (n = 76)	I’m Healthy (n = 38)	I Am Anxious about the Results. (n = 27)	Because I Did not Know about Cancer Screening. (n = 15)	Because I Never had a Chance to Have a Cancer Screening. (n = 184)	Because I Forgot to Take the Test. (n = 57)	I don’t Think I Am Old Enough to Have a Checkup. (n = 58)	Too Much Trouble. (n = 2)	Others (n = 38)
Age										
20–24	81 (16.4%)	8 (9.9%)	3 (3.7%)	0 (0.0%)	5 (6.2%)	23 (28.4%)	7 (8.6%)	32 (39.5%)	0 (0.0%)	3 (3.7%)
25–29	163 (32.9%)	22 (13.5%)	9 (5.5%)	14 (8.6%)	8 (4.9%)	73 (44.8%)	12 (7.4%)	16 (9.8%)	1 (0.6%)	8 (4.9%)
30–34	116 (23.4%)	23 (19.8%)	12 (10.3%)	7 (6.0%)	2 (1.7%)	42 (36.2%)	14 (12.1%)	5 (4.3%)	1 (0.9%)	10 (8.6%)
35–39	135 (27.3%)	23 (17%)	14 (10.4%)	6 (4.4%)	0 (0.0%)	46 (34.1%)	24 (17.8%)	5 (3.7%)	0 (0.0%)	17 (12.6%)
Employment status										
Self-employed	12 (2.4%)	3 (25.0%)	1 (8.3%)	0 (0.0%)	0 (0.0%)	6 (50.0%)	2 (16.7%)	0 (0.0%)	0 (0.0%)	0 (0.0%)
Regular employment	201 (40.6%)	41 (20.4%)	13 (6.5%)	11 (5.5%)	8 (4.0%)	81 (40.3%)	15 (7.5%)	15 (7.5%)	1 (0.5%)	16 (8.0%)
Parttime job	103 (20.8%)	17 (16.5%)	11 (10.7%)	8 (7.8%)	2 (1.9%)	37 (35.9%)	10 (9.7%)	8 (7.8%)	1 (1.0%)	9 (8.7%)
Students	41 (8.3%)	2 (4.9%)	1 (2.4%)	0 (0.0%)	4 (9.8%)	9 (22.0%)	3 (7.3%)	21 (51.2%)	0 (0.0%)	1 (2.4%)
Housewife	100 (20.2%)	10 (10.0%)	11 (11.0%)	4 (4.0%)	0 (0.0%)	43 (43.0%)	18 (18.0%)	7 (7.0%)	0 (0.0%)	7 (7.0%)
Unemployed	38 (7.7%)	3 (7.9%)	1 (2.6%)	4 (10.5%)	1 (2.6%)	8 (21.1%)	9 (23.7%)	7 (18.4%)	0 (0.0%)	5 (13.2%)
Medical insurance *										
Association health insurance	167 (33.7%)	26 (15.6%)	13 (7.8%)	8 (4.8%)	4 (2.4%)	74 (44.3%)	15 (9.0%)	14 (8.4%)	0 (0.0%)	13 (7.8%)
Union health insurance	62 (12.5%)	6 (9.7%)	1 (1.6%)	3 (4.8%)	2 (3.2%)	31 (50.0%)	4 (6.5%)	10 (16.1%)	0 (0.0%)	5 (8.1%)
Mutual aid association	39 (7.9%)	7 (17.9%)	5 (12.8%)	3 (7.7%)	2 (5.1%)	8 (20.5%)	5 (12.8%)	6 (15.4%)	0 (0.0%)	3 (7.7%)
National health insurance	169 (34.1%)	28 (16.6%)	16 (9.5%)	8 (4.7%)	6 (3.6%)	52 (30.8%)	27 (16.0%)	19 (11.2%)	1 (0.6%)	12 (7.1%)
National health insurance association	31 (6.3%)	5 (16.1%)	3 (9.7%)	2 (6.5%)	1 (3.2%)	11 (35.5%)	1 (3.2%)	5 (16.1%)	0 (0.0%)	3 (9.7%)
Unknown	17 (3.4%)	2 (11.8%)	0 (0.0%)	3 (17.6%)	0 (0.0%)	5 (29.4%)	3 (17.6%)	2 (11.8%)	0 (0.0%)	2 (11.8%)
Others	10 (2.0%)	2 (20.0%)	0 (0.0%)	0 (0.0%)	0 (0.0%)	3 (30.0%)	2 (20.0%)	2 (20.0%)	1 (10.0%)	0 (0.0%)

* Employee insurance mainly includes “Association health insurance (for employees of small and medium-sized companies and their dependents),” “Union health insurance (for employees of large companies and their dependents),” “Mutual aid association (for public employees and their dependents),” and “National health insurance association (for doctors, construction workers and their dependents)”. Regional insurance includes “National health insurance (for people who are not covered by employee insurance, such as the self-employed and unemployed)”.

**Table 4:** Psychological and personal characteristics affecting participation of CCS.

Parameter	OR	95%CI	*p*-Value
Age			
35–39	Ref.	----	----
20–24	1.74	0.70–4.32	0.230
25–29	2.22	1.37–3.60	0.001
30–34	1.28	0.79–2.08	0.320
Children			
No	Ref.	----	----
Yes	2.21	1.15–4.25	0.018
What to pay attention to for health			
Have regular health checkups			
No	Ref.	----	----
Yes	2.32	1.38–3.90	0.001
HBM			
Seriousness of cancer	0.87	0.66–1.14	0.315
Benefits of cancer screening	1.31	0.99–1.72	0.055
Importance of cancer screening	0.82	0.60–1.10	0.184
Cues to participation in screening	1.33	1.05–1.69	0.020
Susceptibility to cancer	1.20	0.96–1.51	0.117
Barriers to participation at the time of cancer screening	1.40	1.08–1.81	0.012
Barriers to participation before cancer screening	0.32	0.23–0.45	<0.001

**Table 5:** Summary of results and points to consider when recommending medical examinations.

Summary of Results		Age Groups			
		20–24s	25–29s	30–34s	35–39s
Barrier factors (−)	Because I never had a chance to have a cancer screening	×	●	●	●
	I don’t think I am old enough to have a checkup	●	×	×	×
	Barriers to participation before cancer screening	×	●	●	●
Facilitating factors (+)	Individual complete physical examination/hospital visit	●	●	●	×
	Population-based	×	×	×	●
	Children	×	●	●	×
	Have regular health checkups	×	●	●	●
	Cues to participation in screening	●	●	●	×
	Barriers to participation at the time of cancer screening	●	●	●	●
**Measure**		**Age Groups**			
		**20–24s**	**25–29s**	**30–34s**	**35–39s**
A. Increase opportunities for medical checkups by coordinating community and workplace medical checkups.		×	●	●	●
B. Self-collected cytological diagnosis reduces the burden at the time of screening.		×	×	●	●
C. Education and knowledge dissemination in CCS to reduce the burden prior to screening.		●	●	●	●

Contents that can be speculate from the results and considerations are indicated by “●”, and contents that cannot be speculate are indicated by “×”.

## Data Availability

The data are not publicly available due to confidentiality reasons.

## Supplementary Materials

The following supporting information can be downloaded at: https://www.mdpi.com/article/10.3390/curroncol29090494/s1, Table S1: All personal characteristics used in this study; Table S2: The cervical cancer screening participation status and reasons for non-participation; Table S3: 27 variables of the health belief model in this study.
