# Supplementary material for: Factors Affecting the Cervical Cancer Screening Behaviors of Japanese Women in Their 20s and 30s Using a Health Belief Model: A Cross-Sectional Study
Source: Curr Oncol. 2022 Aug 31;29(9):6287–302. doi: 10.3390/curroncol29090494 (PMC9497901; doi:10.3390/curroncol29090494)
Supplement: Supplementary file 1 [file curroncol-29-00494-s001.zip › curroncol-1874119-supplementary.pdf]

Article

Factors Affecting the Cervical Cancer Screening Behaviors of Japanese Women in Their 20s and 30s Using a Health Belief Model: A Cross-Sectional Study

Zhengai Cui, Hiromi Kawasaki, Miwako Tsunematsu, Yingai Cui and Masayuki Kakehashi

Table S1. All personal characteristics used in this study.

Characteristic	Total (*n*=816)
Region	
Hokkaido	32 (3.9%)
Tohoku	60 (7.4%)
Kanto・Koshinetsu	303 (37.1%)
Tokai・Hokuriku	123 (15.1%)
Kinki	130 (15.9%)
Chugoku・Shikoku	70 (8.6%)
Kyushu・Okinawa	98 (12.0%)
Population size	
23 wards of Tokyo or 1,000,000-city	192 (23.5%)
800,00-city	77 (9.4%)
300,000-city	179 (21.9%)
100,000-city	184 (22.5%)
50,00-city	94 (11.5%)
Towns and villages	90 (11.0%)
Age	
20-24	97 (11.9%)
25-29	293 (35.9%)
30-34	211 (25.9%)
35-39	215 (26.3%)
BMI *	
Lean	173 (21.2%)
Normal	563 (69.0%)
Obese	80 (9.8%)
Marital Status	
Married	460 (56.4%)
Single	356 (43.6%)
Children	
Yes	346 (42.4%)
No	470 (57.6%)
Household composition	
Single	145 (17.8%)
2-person household	163 (20.0%)
2-generation family	422 (51.7%)
3-generation family	59 (7.2%)
Others	27 (3.3%)
Employment status	
Self-employed	26 (3.2%)
Regular employment	340 (41.7%)

Parttime job	152 (18.6%)
Students	46 (5.6%)
Housewife	205 (25.1%)
Unemployed	47 (5.8%)
Educational Background	
Primary and secondary school	22 (2.7%)
High School	196 (24.0%)
Junior colleges and vocational schools	174 (21.3%)
University	299 (36.6%)
Graduate School	24 (2.9%)
Others	101 (12.4%)
Household income **	
No income	15 (1.8%)
Less than $7324	23 (2.8%)
$7324-$21898	97 (11.9%)
$21971-$36545	266 (32.6%)
$36618-$51192	211 (25.9%)
$51265-$73229	124 (15.2%)
$73236-$109781	61 (7.5%)
More than $109854	19 (2.3%)
Medical insurance ***	
Association health insurance	319 (39.1%)
Union health insurance	107 (13.1%)
Mutual aid association	75 (9.2%)
National health insurance	233 (28.6%)
National health insurance association	50 (6.1%)
Unknown	20 (2.5%)
Others	12 (1.5%)
Medical insurance (dependent)	
Myself	465 (57.0%)
Family	351 (43.0%)
Family history of breast cancer	
Yes	73 (8.9%)
No	743 (91.1%)
Family history of cervical cancer	
Yes	36 (4.4%)
No	780 (95.6%)
Family history of others cancer	
Yes	336 (41.2%)
No	480 (58.8%)
Medical consultation	
Yes	198 (24.3%)
No	618 (75.7%)
Health Status	
Bad	19 (2.3%)
Slightly bad	123 (15.1%)
Normal	433 (53.1%)
Slightly good	149 (18.3%)
Good	92 (11.3%)
Are you taking care of your own health	
Not careful at all	22 (2.7%)
Not very careful	134 (16.4%)
Cannot say either way	186 (22.8%)

Sometimes very careful	365 (44.7%)
Always very careful	109 (13.4%)
Do you have health concerns	
Very anxious	77 (9.4%)
A little anxious	369 (45.2%)
Cannot say either way	241 (29.5%)
No anxiety	102 (12.5%)
No anxiety at all	27 (3.3%)
What to pay attention to for health	
Pay attention to diet	
Yes	495 (60.7%)
No	321 (39.3%)
Get enough sleep	
Yes	508 (62.3%)
No	308 (37.7%)
Drink too much alcohol	
Yes	288 (35.3%)
No	528 (64.7%)
Smoking cigarettes	
Yes	451 (55.3%)
No	365 (44.7%)
Have regular health checkups	
Yes	136 (16.7%)
No	680 (83.3%)
Do regular exercise	
Yes	183 (22.4%)
No	633 (77.6%)
Avoid stress	
Yes	282 (34.6%)
No	534 (65.4%)
Rinse your mouth and wash your hands	
Yes	384 (47.1%)
No	432 (52.9%)
Others	
Yes	12 (1.5%)
No	804 (98.5%)
The most feared disease	
Cancer	336 (41.2%)
Heart disease	51 (6.3%)
Brain Attack	105 (12.9%)
Pneumonia	6 (0.7%)
Diabetes	54 (6.6%)
Liver disease	10 (1.2%)
Dementia	83 (10.2%)
Depression	53 (6.5%)
None	104 (12.7%)
Others	14 (1.7%)
Influenza vaccination	
Vaccinated every year	218 (26.7%)
Sometimes vaccinated	144 (17.6%)
Vaccinated by chance	49 (6.0%)
Not vaccinated	306 (37.5%)
Thinking vaccination is useless	99 (12.1%)

Private medical insurance	
Yes	400 (49.0%)
No	416 (51.0%)

**BMI: Lean (BMI<18.5), Normal(18.5≤BMI<25), and Obese (BMI≥25). **Household income: Calculated according to the exchange rate on July 26, 2022 (1 yen = $0.0073). ***Employee insurance mainly includes "Association health insurance (for employees of small and medium-sized companies and their dependents)," "Union health insurance (for employees of large companies and their dependents)," "Mutual aid association (for public employees and their dependents)," and "National health insurance association (for doctors, construction workers and their dependents). Regional insurance includes "National health insurance (for people who are not covered by employee insurance, such as the self-employed and unemployed).

Table S2. The cervical cancer screening participation status and reasons for non-participation.

**Characteristic**	**Total (*n*=816)**
Participation in cervical cancer screening	
Yes	321 (39.3%)
No	495 (60.7%)
Status of participation in cervical cancer screening	
Population-based	127 (39.6%)
Workplace-based	42 (13.1%)
Individual complete physical examination/hospital visit	137 (42.7%)
Others	15 (4.7%)
Reasons for not participating in cervical cancer screening	
Busy	76 (15.4%)
I'm healthy	38 (7.7%)
I am anxious about the results	27 (5.5%)
Because I did not know about cancer screening	15 (3.0%)
Because I never had a chance to have a cancer screening	184 (37.2%)
Because I forgot to take the test	57 (11.5%)
I don't think I am old enough to have a checkup	58 (11.7%)
Too much trouble	2 (0.4%)
Others	38 (7.7%)

Table S3. 27 variables of the health belief model in this study.

**Characteristic**	**Strongly disagree**	**Disagree**	**Neither agree nor disagree**	**Agree**	**Strongly agree**
I may develop female cancer in the future.	31 (3.8%)	79 (9.7%)	374 (45.8%)	265 (32.5%)	67 (8.2%)
I may develop female cancer within a few years.	61 (7.5%)	181 (22.2%)	496 (60.8%)	60 (7.4%)	18 (2.2%)
I am more likely to develop female cancer than other women.	58 (7.1%)	176 (21.6%)	436 (53.4%)	123 (15.1%)	23 (2.8%)
I am afraid of developing female cancer.	10 (1.2%)	15 (1.8%)	60 (7.4%)	360 (44.1%)	371 (45.5%)
If I develop female cancer, my life will be changed.	4 (0.5%)	22 (2.7%)	100 (12.3%)	364 (44.6%)	326 (40.0%)
If I develop female cancer, my activities of daily living will be limited.	3 (0.4%)	31 (3.8%)	100 (12.3%)	377 (46.2%)	305 (37.4%)
If I develop female cancer, my family will be made trouble.	8 (1.0%)	29 (3.6%)	78 (9.6%)	369 (45.2%)	332 (40.7%)
I'm scared that I'll find a woman's cancer by having a cancer screening.	31 (3.8%)	103 (12.6%)	153 (18.8%)	306 (37.5%)	223 (27.3%)

Participation in female cancer screening can lead to reduction in mortality from female cancer.	7 (0.9%)	30 (3.7%)	107 (13.1%)	379 (46.5%)	293 (35.9%)
Participation in female cancer screening can lead to early detection of female cancer.	3 (0.4%)	7 (0.9%)	53 (6.5%)	352 (43.1%)	401 (49.1%)
Having a women's cancer screening can give you peace of mind about your health.	7 (0.9%)	19 (2.3%)	107 (13.1%)	410 (50.3%)	273 (33.5%)
Participation in female cancer screening can lead to better management of my health.	9 (1.1%)	8 (1.0%)	85 (10.4%)	424 (52.0%)	290 (35.5%)
I do not have time to participate in female cancer screening.	58 (7.1%)	185 (22.7%)	235 (28.8%)	254 (31.1%)	84 (10.3%)
Participation in female cancer screening is costly.	14 (1.7%)	88 (10.8%)	156 (19.1%)	368 (45.1%)	190 (23.3%)
I don't know where to go for a female cancer screening.	138 (16.9%)	240 (29.4%)	154 (18.9%)	204 (25.0%)	80 (9.8%)
I forget to regularly participate in female cancer screening.	37 (4.5%)	142 (17.4%)	225 (27.6%)	319 (39.1%)	93 (11.4%)
I am embarrassed about participating in female cancer screening because it includes examination of a delicate area.	81 (9.9%)	174 (21.3%)	171 (21.0%)	251 (30.8%)	139 (17.0%)
Female cancer screening causes discomfort, even pain.	19 (2.3%)	98 (12.0%)	239 (29.3%)	313 (38.4%)	147 (18.0%)
I do not want to participate in female cancer screening that is performed by male doctors/staff members.	57 (7.0%)	154 (18.9%)	225 (27.6%)	224 (27.5%)	156 (19.1%)
There are other things in my life that are more important than getting screened for women's cancer.	63 (7.7%)	226 (27.7%)	385 (47.2%)	106 (13.0%)	36 (4.4%)
I do not need to participate in female cancer screening because I can visit a medical institution whenever there is any concern.	121 (14.8%)	371 (45.5%)	269 (33.0%)	42 (5.2%)	13 (1.6%)
I do not need to participate in female cancer screening because I have no subjective symptoms.	136 (16.7%)	368 (45.1%)	212 (26.0%)	86 (10.5%)	14 (1.7%)
Participation in female cancer screening is less important than other health issues.	135 (16.5%)	412 (50.5%)	222 (27.2%)	37 (4.5%)	10 (1.2%)
A close friend or acquaintance recommends that you have a cancer screening for women.	121 (14.8%)	211 (25.9%)	303 (37.1%)	155 (19.0%)	26 (3.2%)
My closest family members recommend that I participate in female cancer screening.	117 (14.3%)	222 (27.2%)	255 (31.3%)	171 (21.0%)	51 (6.3%)
The doctors at the hospital which I regularly visit recommend that I participate in female cancer screening.	192 (23.5%)	277 (34.0%)	238 (29.2%)	95 (11.6%)	14 (1.7%)
My close friends/acquaintances recommend that I participate in female cancer screening.	78 (9.6%)	152 (18.6%)	327 (40.1%)	214 (26.2%)	45 (5.5%)
